# Feasibility and Prognostic Value of an Ovarian NAR-like Score in Advanced Ovarian Cancer Treated with Neoadjuvant Chemotherapy and Interval Debulking Surgery

**DOI:** 10.3390/jcm15166266

**Published:** 2026-08-13

**Authors:** Yeşim Özkaya Uçar, Arife Ebru Kuzu, Okan Aytekin, Serhat Sekmek, Safa Can Efil, Mehmet Ünsal, Fatih Kılıç, Taner Turan

**Affiliations:** 1 Department of Gynecologic Oncology, Ankara Bilkent City Hospital, University of Health Sciences, 06800 Ankara, Turkey; 2 Department of Medical Oncology, Ankara Bilkent City Hospital, 06800 Ankara, Turkey

**Keywords:** ovarian cancer, neoadjuvant chemotherapy, interval debulking surgery, neoadjuvant rectal score, oNAR, progression-free survival, prognostic score

## Abstract

**Background**: Neoadjuvant chemotherapy followed by interval debulking surgery is an established treatment strategy for selected patients with advanced epithelial ovarian cancer. However, simple postoperative prognostic tools integrating pre-treatment disease extent and post-treatment pathological stage remain limited. The neoadjuvant rectal (NAR) score was originally developed as a composite stage-migration endpoint in rectal cancer and has subsequently been explored in other solid tumors. We evaluated the feasibility and prognostic value of an ovarian NAR-like score (oNAR) in patients with advanced ovarian cancer treated with neoadjuvant chemotherapy and interval debulking surgery. **Methods**: This single-center retrospective cohort included patients with advanced ovarian cancer treated with platinum-taxane-based neoadjuvant chemotherapy followed by interval debulking surgery. The oNAR score was calculated using clinical T category before neoadjuvant chemotherapy and pathological T and N categories after interval surgery: oNAR = [5 × ypN − 3 × (cT − ypT) + 12]^2^/9.61. The primary analysis evaluated oNAR as a continuous score. Low, intermediate, and high oNAR categories based on the original rectal NAR thresholds were used only for exploratory visualization. The primary endpoint was progression-free survival (PFS). Cox regression, Kaplan–Meier analysis, log-rank testing, Harrell concordance index, and exploratory ROC analysis were used. **Results**: Among 74 reviewed records, 73 patients had sufficient cT, ypT, and ypN data for oNAR calculation and were included in the primary analysis. Thirty-one PFS events and 10 deaths occurred. The median oNAR was 14.98 (IQR, 14.98–30.07). As a continuous variable, higher oNAR was associated with shorter PFS (HR per 1-point increase, 1.036; 95% CI, 1.010–1.061; *p* = 0.0055), corresponding to an HR of 1.42 per 10-point increase. The Harrell C-index for PFS was 0.669. In a parsimonious comparison, oNAR showed higher discrimination than ypN alone and discrimination comparable to a combined ypT/ypN model. PFS differed significantly across low-, intermediate-, and high-oNAR groups (log-rank *p* = 0.0053). Median PFS was not reached in the low-oNAR group, compared with 13.47 months in the intermediate group and 12.09 months in the high group. The association persisted in sensitivity analyses restricted to patients with complete/optimal cytoreduction, maximal cytoreduction, and high-grade serous histology. **Conclusions**: The oNAR score was feasible to calculate using routinely available clinical and pathological staging variables and was significantly associated with PFS after neoadjuvant chemotherapy and interval debulking surgery. These findings support oNAR as a feasible stage-migration-based prognostic summary that may help capture post-NACT pathological T/N information and warrants external validation in larger ovarian cancer cohorts.

## 1. Introduction

Epithelial ovarian cancer remains one of the most lethal gynecologic malignancies because many patients present with advanced intraperitoneal disease [1]. In advanced-stage disease, treatment is centered on maximal cytoreductive surgery and platinum-taxane-based systemic therapy. Contemporary guidelines support either primary cytoreductive surgery or neoadjuvant chemotherapy (NACT) followed by interval debulking surgery (IDS), depending on disease distribution, expected resectability, patient fitness, and multidisciplinary assessment [2,3].

Randomized trials established NACT followed by IDS as an acceptable strategy in selected patients with stage IIIC-IV ovarian, fallopian tube, or primary peritoneal carcinoma [4,5]. Subsequent pooled and trial-based evidence confirmed that this approach can achieve comparable survival in appropriately selected patients while modifying surgical invasiveness and perioperative morbidity profiles [3,6]. Across treatment strategies, complete cytoreduction remains one of the most consistent determinants of outcome in advanced ovarian cancer [7,8].

Risk stratification after NACT remains clinically important but incompletely standardized. Histopathologic chemotherapy response score (CRS) has been validated in high-grade serous tubo-ovarian carcinoma, particularly using omental response assessment [9,10]. Serum CA-125 kinetics and KELIM provide complementary information on chemosensitivity and surgical outcome, but they require serial measurements and specific modeling [11,12].

The neoadjuvant rectal (NAR) score was developed in rectal cancer as a short-term composite endpoint based on clinical T stage, post-treatment pathological T stage, and post-treatment nodal status [13,14]. The clinical relevance of the NAR score lies in its ability to convert treatment-related stage migration into a single, reproducible prognostic measure. Rather than relying only on final pathological stage, NAR incorporates both pretreatment clinical extent and post-treatment pathological response, thereby providing a compact summary of tumor downstaging and residual disease burden after neoadjuvant therapy. This concept may be particularly applicable to ovarian cancer treated with NACT followed by IDS, where both baseline disease extent and residual pathological disease after chemotherapy are clinically relevant, but are not routinely integrated into a single quantitative score. The attraction of this approach is its simplicity: it summarizes treatment-associated stage migration using variables routinely available before and after neoadjuvant therapy. Similar NAR-based adaptations have been explored in gastric and breast cancer, suggesting that stage-migration concepts may be transferable across solid tumors [15,16].

To our knowledge, a NAR-like score has not been systematically evaluated in ovarian cancer treated with NACT and IDS. We therefore evaluated the feasibility and prognostic value of an ovarian NAR-like score (oNAR) calculated from pre-NACT cT and post-NACT ypT/ypN. To avoid overlap with pretreatment inflammatory, nutritional, or comorbidity-based prognostic marker studies, this analysis deliberately focused on post-treatment stage migration and PFS.

## 2. Materials and Methods

### 2.1. Study Design and Population

This was a single-center retrospective cohort study of patients with advanced ovarian cancer treated with neoadjuvant chemotherapy (NACT) followed by interval debulking surgery (IDS). The study reviewed the data of 74 patients with advanced ovarian cancer who underwent IDS after NACT at the Gynecologic Oncology Clinic of Ankara Bilkent City Hospital. Among these patients, 73 had sufficient clinical and pathological staging data for calculation of the ovarian NAR-like score (oNAR) and constituted the primary analysis cohort.

Eligible patients were women with epithelial ovarian, fallopian tube, or primary peritoneal carcinoma who received platinum-taxane-based NACT and subsequently underwent IDS. For the oNAR analysis, patients were required to have available pre-NACT clinical T category and post-NACT pathological T and N categories. Patients without sufficient staging data to calculate oNAR were excluded from the primary analysis. The present study focused specifically on stage migration and did not re-evaluate pretreatment inflammatory, nutritional, or comorbidity-based prognostic indices. Patients with FIGO stage IV disease were considered for IDS only if they had adequate performance status, radiological and/or clinical response to NACT, and disease considered surgically cytoreducible by the multidisciplinary team. Therefore, the study cohort represents a selected population of stage IV patients who proceeded to interval surgery after NACT, rather than an unselected stage IV ovarian cancer population.

The study was approved by the Ankara Bilkent City Hospital No. 1 Medical Research Scientific and Ethics Evaluation Committee (TABED 1-26-2516; approval date: 22 April 2026). The study was conducted in accordance with the principles of the Declaration of Helsinki. Because of the retrospective design and use of anonymized institutional data, the requirement for informed consent was handled according to the decision of the local ethics committee.

### 2.2. Treatment and Pathological Assessment

NACT consisted predominantly of carboplatin plus paclitaxel according to institutional practice. The number of NACT cycles and timing of IDS were determined by the treating team based on clinical response, imaging findings, performance status, and multidisciplinary evaluation.

Pre-NACT cT and cN categories were abstracted from pre-treatment imaging, operative assessment when available, and institutional staging records. FIGO stage IV disease was defined from the baseline metastatic/stage variable rather than from isolated M-code entries, because some historical records used free-text metastatic-site documentation. Post-NACT ypT and ypN categories were abstracted from final pathology reports after IDS. For the purpose of oNAR calculation, T substages were collapsed into their main numerical T categories: T1a–T1c were coded as 1, T2a–T2b as 2, and T3a–T3c as 3. Nodal status was coded as 0 for node-negative disease and 1 for node-positive disease. Cytoreduction was recorded according to institutional surgical documentation but was not used as a defining component of oNAR.

### 2.3. Definition of the Ovarian NAR-like Score

The oNAR score was adapted from the original NAR formula and calculated as follows:

oNAR = [5 × ypN − 3 × (cT − ypT) + 12]^2^/9.61

In this formula, cT indicates the pre-NACT clinical T category, ypT indicates the post-NACT pathological T category, and ypN indicates post-NACT pathological nodal status. The denominator 9.61 was retained from the original NAR formulation to preserve the structure and scaling of the previously described score. Because this constant was not derived from ovarian cancer cohorts, we performed sensitivity analyses using standardized and rescaled oNAR values to evaluate whether the prognostic findings were dependent on the original denominator. Because ovarian cancer staging is not identical to rectal cancer staging, and because the relative prognostic weight of T and N categories may differ between tumor types, this adaptation was considered exploratory and hypothesis-generating.

The primary analysis evaluated oNAR as a continuous score. Low-, intermediate-, and high-oNAR categories were defined a priori according to the original rectal NAR thresholds, as used in previous NAR-based adaptations, to facilitate descriptive interpretation and avoid data-driven cut-point selection. Although these predefined categories significantly stratified PFS in the present cohort, their clinical use as ovarian cancer-specific risk thresholds requires external validation.

### 2.4. Endpoints

The primary endpoint was progression-free survival (PFS), defined as the interval from IDS to radiological, biochemical, or clinical progression/recurrence, death, or last follow-up, according to the available institutional follow-up data. The secondary endpoint was overall survival (OS), defined as the interval from IDS to death or last follow-up. OS analyses were interpreted cautiously because of the low number of death events.

### 2.5. Statistical Analysis

Continuous variables were summarized as median and interquartile range (IQR), and categorical variables as number and percentage. Between-group comparisons were descriptive; *p* values for oNAR-defining variables were not interpreted as independent hypothesis tests because cT, ypT, and ypN are mathematical components of the score.

The association between oNAR and PFS was evaluated using Cox proportional hazards regression. oNAR was assessed as a continuous variable per 1-point and per 10-point increase. Kaplan–Meier curves were generated for low-, intermediate-, and high-oNAR groups and compared with the log-rank test. An adjusted Cox model included oNAR, age, and FIGO stage IV status. Because of the number of PFS events, multivariable modeling was intentionally limited to avoid overfitting. An exploratory model evaluated whether oNAR retained significance after adjustment for log-transformed preoperative CA-125.

Given the limited number of PFS events, we also performed a predefined parsimonious comparison of oNAR with residual nodal disease alone and with post-NACT pathological T/N status. Specifically, PFS association and discrimination were evaluated for oNAR as a continuous score, ypN status, ypT category, and a combined ypT/ypN Cox model. These analyses were performed to determine whether the prognostic signal of oNAR was mainly attributable to residual nodal disease alone or to the combined post-treatment pathological T/N status.

Discrimination was assessed using Harrell concordance index for PFS and exploratory ROC analysis for binary recurrence/progression status [17]. C-index results were interpreted as global discrimination rather than as evidence of clinical calibration. Sensitivity analyses were performed in patients with complete/optimal cytoreduction, in patients with maximal cytoreduction, and in patients with high-grade serous histology. Two-sided *p* values < 0.05 were considered statistically significant. Statistical analyses were performed using IBM SPSS Statistics, version 26.0 (IBM Corp., Armonk, NY, USA).

## 3. Results

### 3.1. Patient Selection and Feasibility of oNAR Calculation

**Figure 1:**
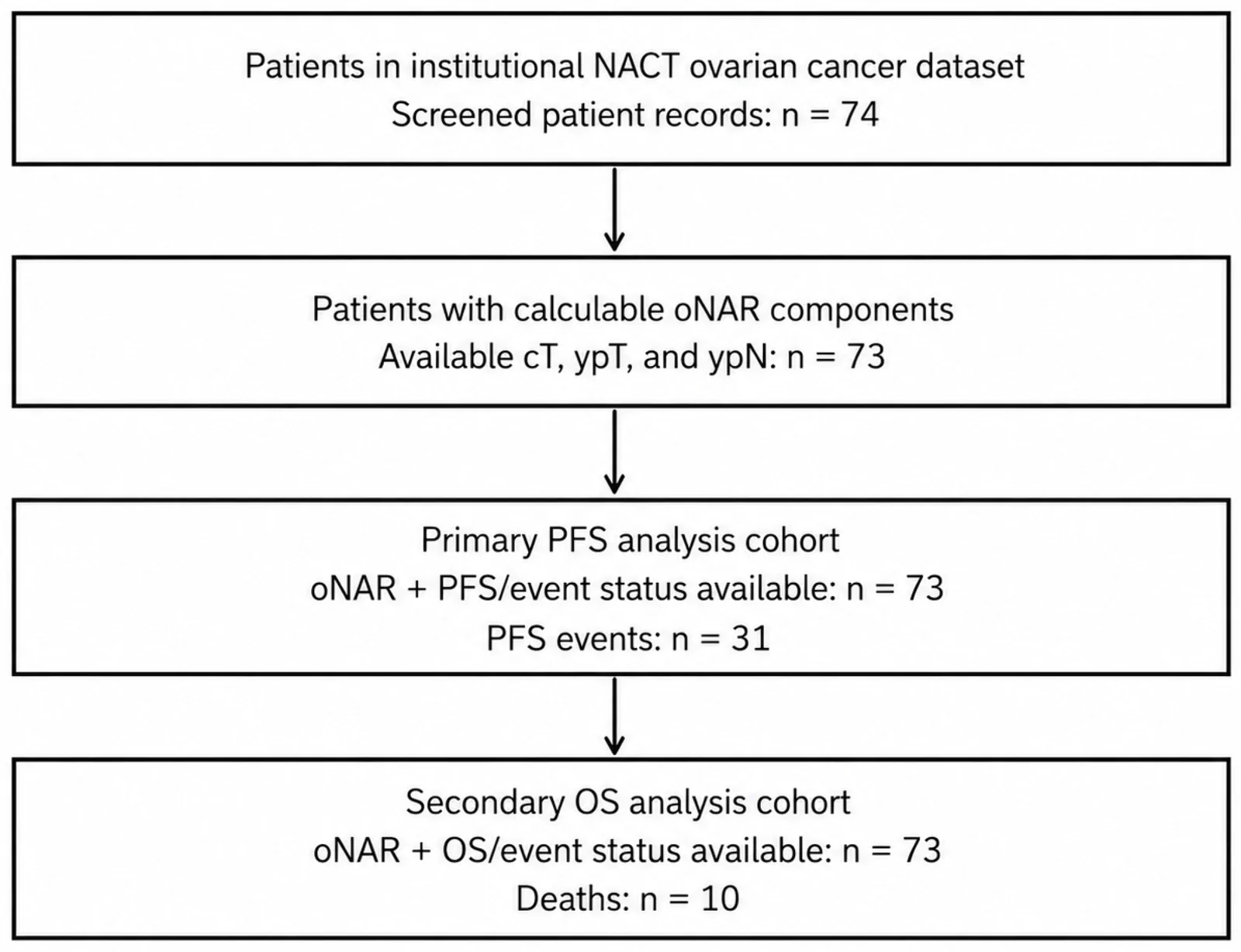
Study flow diagram and oNAR data availability.

A total of 74 non-empty patient records were reviewed. Sufficient cT, ypT, and ypN data were available for 73 patients, who formed the primary oNAR cohort. Missingness was limited for the primary variables: cT was available for all reviewed patients, whereas ypT and ypN were each missing in one patient. CRS was available only in a minority of patients and was therefore not included in the primary model. Figure 1 summarizes patient selection and data availability.

### 3.2. Baseline and Stage Characteristics According to oNAR Group

The oNAR cohort included 16 patients in the low-oNAR group, 33 in the intermediate-oNAR group, and 24 in the high-oNAR group. Age and FIGO stage IV disease were broadly similar across oNAR groups. For descriptive consistency, FIGO stage IV disease was derived from the baseline metastatic/stage variable, which identified 36 patients with metastatic disease at diagnosis. As expected, ypT and ypN differed across oNAR categories because these variables define the score. All high-oNAR patients had ypN-positive disease after IDS. Among FIGO stage IV patients, interval surgery was performed after selection based on response to NACT and multidisciplinary assessment of resectability. Stage IV classification was most commonly related to distant nodal involvement, pleural disease/effusion, or limited extra-abdominal/metastatic involvement rather than uniformly unresectable disseminated disease (Table 1 and Table 2).

### 3.3. Progression-Free Survival According to oNAR

Thirty-one PFS events occurred among the 73 patients included in the primary analysis. Median oNAR was 14.98 (IQR, 14.98–30.07). When analyzed as a continuous variable, higher oNAR was significantly associated with shorter PFS (HR per 1-point increase, 1.036; 95% CI, 1.010–1.061; *p* = 0.0055). This corresponded to an HR of 1.42 for each 10-point increase in oNAR (95% CI, 1.108–1.815; *p* = 0.0055). The Harrell C-index for oNAR in relation to PFS was 0.669.

**Figure 2:**
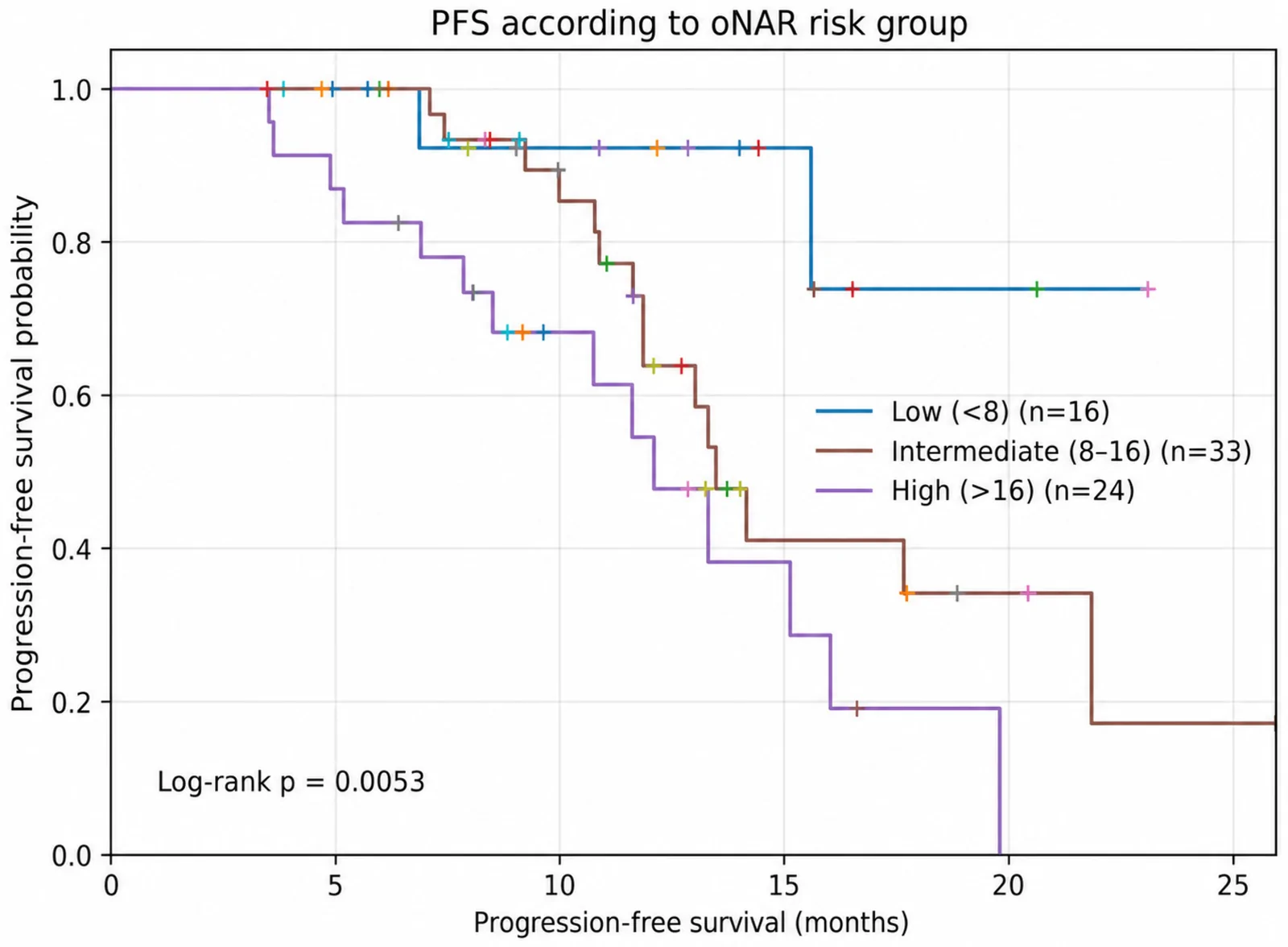
PFS according to oNAR risk group. The “+” symbols indicate censored observations.

Kaplan–Meier analysis showed clear separation of PFS across oNAR groups (log-rank *p* = 0.0053). Median PFS was not reached in the low-oNAR group, whereas it was 13.47 months in the intermediate-oNAR group and 12.09 months in the high-oNAR group. The 12-month PFS rates were 92.3%, 63.8%, and 54.5%, respectively; the corresponding 18-month PFS rates were 73.8%, 34.2%, and 19.1% (Figure 2 and Table 3).

### 3.4. Parsimonious Comparison of oNAR with ypN and ypT/ypN

In the predefined parsimonious comparison, ypN positivity alone was associated with shorter PFS (HR, 2.654; 95% CI, 1.288–5.471; *p* = 0.0082), but its discrimination was lower than that of oNAR as a continuous score (C-index, 0.630 vs. 0.669). The ypT category was also associated with PFS (HR per category increase, 2.076; 95% CI, 1.140–3.780; *p* = 0.0170; C-index, 0.616). A combined ypT/ypN model achieved discrimination similar to oNAR (C-index, 0.678 vs. 0.669), indicating that the prognostic information captured by oNAR is closely related to post-NACT pathological T/N status. Therefore, oNAR should be interpreted as a compact stage-migration-based composite score rather than as an independent replacement for pathological staging (Table 4).

### 3.5. Exploratory and Sensitivity Analyses

Exploratory ROC analysis for binary recurrence/progression status yielded an AUC of 0.682. Because the primary endpoint was time-to-event PFS and because the study was not designed to develop a new binary prediction model, ROC findings were not used to select a new cut-off and are reported only as exploratory discrimination data.

Sensitivity analyses using standardized and rescaled oNAR values yielded unchanged discrimination and materially similar associations with PFS, supporting the interpretation that the denominator functions primarily as a scaling constant rather than as an ovarian cancer-specific prognostic weight.

The association between oNAR and PFS remained directionally consistent in sensitivity analyses restricted to patients with complete/optimal cytoreduction, patients with maximal cytoreduction only, and patients with high-grade serous histology. These findings support the consistency of the primary PFS signal, but they should be interpreted cautiously because subgroup sample sizes and event numbers were limited.

## 4. Discussion

In this retrospective cohort of patients with advanced ovarian cancer treated with NACT and IDS, an ovarian NAR-like score was feasible to calculate in nearly all reviewed records with available staging information. Higher oNAR was significantly associated with shorter PFS both as a continuous score and when categorized into low, intermediate, and high groups. The association persisted after adjustment for age and FIGO stage IV status and remained directionally consistent in clinically relevant sensitivity cohorts. These findings suggest that a simple stage-migration-based score derived from pre-treatment clinical T category and post-treatment pathological T/N categories may provide prognostic information after NACT and IDS.

The main clinical implication of this study is that treatment-associated stage migration can be summarized using a score based on routinely available staging variables. Unlike biomarkers requiring serial laboratory modeling or specialized pathological scoring, oNAR can be calculated from information commonly present in pre-NACT staging records and IDS pathology reports. This may be particularly useful for retrospective datasets and for centers where standardized CRS documentation has not been uniformly implemented. However, oNAR should be interpreted as an exploratory prognostic summary rather than as a definitive clinical prediction model.

The present study builds on the conceptual framework of the NAR score in rectal cancer, where clinical T stage, post-treatment pathological T stage, and nodal response were combined into a composite metric intended to reflect the effect of neoadjuvant therapy [18,19]. Subsequent adaptations in gastric and breast cancer suggest that NAR-like approaches may have broader relevance when pre-treatment tumor burden and post-treatment pathological response are both available [15,16,20,21]. Ovarian cancer differs substantially from rectal cancer in terms of anatomy, patterns of spread, surgical endpoints, and biological behavior. Therefore, direct transfer of the original NAR formula to ovarian cancer must be interpreted cautiously. Nevertheless, the observed association between oNAR and PFS suggests that the core concept of stage-migration-based risk stratification may be applicable after NACT and IDS.

The revised parsimonious comparison clarified that the prognostic signal of oNAR was closely related to post-NACT pathological T/N status. This finding was expected to some extent because ypN is an explicit component of the score and because baseline cT categories were relatively homogeneous in this cohort. The influence of residual nodal disease does not invalidate oNAR; rather, it reflects the biological and prognostic relevance of persistent nodal involvement after NACT. Nevertheless, oNAR should not be interpreted as independent of ypN or as a replacement for detailed pathological staging. Instead, it may be regarded as a compact stage-migration-based summary score that showed higher discrimination than ypN alone and discrimination comparable to a combined ypT/ypN model. Our findings should be viewed alongside existing response assessment tools in advanced ovarian cancer. CRS is a validated histopathological marker in high-grade serous tubo-ovarian carcinoma, particularly in omental response assessment [22,23]. Several meta-analyses and contemporary reviews have further supported its prognostic value, although heterogeneity across cohorts and differences in implementation remain important considerations [24,25]. In addition, recent data suggest that the prognostic performance of CRS may be influenced by contemporary treatment factors such as BRCA status and maintenance therapy exposure [26]. Furthermore, the expanding role of targeted therapies, including antibody–drug conjugates in gynecologic cancers, underscores the need to validate historical stage- and response-based prognostic tools within contemporary molecularly guided treatment settings [27]. CA-125 kinetics and KELIM also provide valuable information on chemosensitivity and surgical outcome [12,13]. oNAR should not be considered a replacement for CRS, CA-125 kinetics, KELIM, molecular markers, or residual disease assessment. Rather, it may complement these tools by providing a simple stage-based summary of post-NACT residual disease.

The exploratory model combining oNAR with preoperative CA-125 suggested that both variables may capture different dimensions of prognosis. oNAR reflects anatomical stage migration and residual pathological disease, whereas CA-125 may reflect tumor activity, disease burden, and chemosensitivity. However, because of the limited cohort size and number of events, this combined model should be interpreted as hypothesis-generating. The present study was not designed to develop a definitive multivariable prediction model or to establish a clinically actionable cut-off beyond the predefined NAR-based categories.

This study has several limitations. First, it was retrospective and single-center, which may limit its generalizability. The high rate of adequate cytoreduction among FIGO stage IV patients likely reflects selection for IDS after response to NACT and multidisciplinary assessment of surgical resectability, and should therefore not be generalized to all patients with stage IV ovarian cancer. Second, the cohort size was modest, and the number of OS events was low; therefore, PFS was selected as the primary endpoint and OS was interpreted cautiously. Third, the baseline clinical T stage was relatively homogeneous, limiting the independent contribution of pretreatment T category and increasing the relative influence of post-treatment pathological T and nodal status on the score. Fourth, residual disease after cytoreduction is a major prognostic factor in advanced ovarian cancer, but it was not incorporated into the oNAR formula because the score was deliberately designed as a stage-migration-based metric. Although sensitivity analyses according to cytoreduction status were directionally consistent, the sample size limited more extensive multivariable adjustment. Fifth, CRS data were missing in a large proportion of patients and were not included in the primary model. Sixth, the oNAR formula was adapted from rectal cancer and has not yet been externally validated in ovarian cancer. Although sensitivity analyses suggested that the denominator mainly affected score scaling rather than prognostic discrimination, the optimal ovarian cancer-specific weighting and scaling of the oNAR formula should be evaluated in larger external cohorts. Similarly, although the predefined low-, intermediate-, and high-oNAR categories significantly stratified PFS in this cohort, they were derived from the original rectal NAR framework and require external validation before being used as ovarian cancer-specific clinical risk thresholds. Finally, calibration, external validation, comparison with established prognostic models, and assessment of incremental value beyond ypT, ypN, CRS, residual disease, and molecular variables were beyond the scope of this initial feasibility study.

Despite these limitations, this study has several strengths. The analysis used a predefined, literature-based formula and avoided data-driven cut-point selection for the main risk groups. It focused on a clearly defined NACT-IDS population and deliberately avoided overlap with pretreatment inflammatory or nutritional marker analyses. The primary endpoint was time-to-event PFS, and sensitivity analyses supported the consistency of the association across clinically relevant subgroups. In addition, the score was feasible to calculate in almost all reviewed records, supporting its potential utility in retrospective datasets and real-world clinical cohorts.

In conclusion, oNAR is a feasible, simple, and routinely calculable stage-migration-based prognostic score in advanced ovarian cancer treated with NACT and IDS. Higher oNAR was significantly associated with shorter PFS. These findings support external validation of oNAR in larger multicenter cohorts and prospective datasets, ideally alongside CRS, CA-125 kinetics, BRCA/HRD status, maintenance treatment exposure, residual disease metrics, and standardized pathological response assessment.

## Figures and Tables

**Table 1:** Baseline and oNAR-relevant staging variables according to oNAR group.

Variable	Low oNAR (<8) (*n* =16)	Intermediate oNAR (8–16) (*n* = 33)	High oNAR (>16) (*n* = 24)	*p* Value
Age, years	60.3 (55.7–68.7)	59.3 (52.6–67.8)	61.0 (56.0–65.6)	0.892
FIGO stage IV	8/16 (50.0%)	15/33 (45.5%)	13/24 (54.2%)	0.808
cT category	cT3: 16	cT1: 1; cT3: 32	cT1: 1; cT3: 23	0.725
cN positive	6/16 (37.5%)	12/33 (36.4%)	18/24 (75.0%)	0.009
ypT category	ypT0: 7; ypT1: 9	ypT1: 1; ypT2: 2; ypT3: 30	ypT3: 24	<0.001
ypN positive	0/16 (0.0%)	0/33 (0.0%)	24/24 (100.0%)	<0.001
NACT cycles	3.0 (3.0–4.0)	3.0 (3.0–4.0)	3.0 (3.0–4.0)	0.876

Values are median (IQR) or *n*/N (%). *p* values are descriptive. cT—clinical T before NACT; cN—clinical N before NACT; ypT/ypN—pathological T/N after IDS. Variables defining oNAR are expected to differ across oNAR groups and should not be interpreted as independent baseline differences. NACT—neoadjuvant chemotherapy; IDS—interval debulking surgery; oNAR—ovarian NAR-like score; FIGO—International Federation of Gynecology and Obstetrics; IQR—interquartile range.

**Table 2:** oNAR components and risk groups in the primary cohort.

oNAR Variable	Primary Cohort (*n* = 73)
cT before NACT	cT1: 2; cT3: 61; cT3c: 10
cN before NACT	cN0: 37; cN1: 36
FIGO stage IV disease at diagnosis	36/73 (49.3%)
ypT after IDS	ypT0: 7; ypT1/1a/1b/1c: 10; ypT2/2a: 2; ypT3/3b/3c: 54
ypN after IDS	ypN0: 49; ypN1: 24
oNAR risk group	Low (<8): 16; Intermediate (8–16): 33; High (>16): 24

cT/cN—clinical T/N categories before NACT; ypT/ypN—pathological T/N categories after IDS. FIGO stage IV disease was based on the baseline metastatic/stage variable. For oNAR calculation, T substages were collapsed into the main numerical T category; therefore, cT3c and ypT3b/c were coded as T3. NACT—neoadjuvant chemotherapy; IDS—interval debulking surgery; FIGO—International Federation of Gynecology and Obstetrics; oNAR—ovarian NAR-like score.

**Table 3:** PFS and OS outcomes according to oNAR group.

oNAR Group	*n*	oNAR Median (IQR)	PFS Events, *n* (%)	Median PFS, Months	12-Month PFS	18-Month PFS	24-Month PFS	OS Events, *n* (%)	Median OS, Months
Low (<8)	16	3.75 (0.94–3.75)	2 (12.5%)	NR	92.3%	73.8%	73.8%	1 (6.2%)	NR
Intermediate (8–16)	33	14.98 (14.98–14.98)	15 (45.5%)	13.47	63.8%	34.2%	17.1%	4 (12.1%)	NR
High (>16)	24	30.07 (30.07–30.07)	14 (58.3%)	12.09	54.5%	19.1%	0.0%	5 (20.8%)	NR

NR—not reached; PFS—progression-free survival; OS—overall survival; IQR—interquartile range; oNAR—ovarian NAR-like score.

**Table 4:** Parsimonious comparison of predefined staging-based predictors for progression-free survival.

Predictor/Model	Hazard Ratio	95% CI	*p* Value	Harrell C-Index
oNAR, per 1-point increase	1.036	1.010–1.061	0.0055	0.669
oNAR, per 10-point increase	1.419	1.108–1.815	0.0055	0.669
ypN positive vs. negative	2.654	1.288–5.471	0.0082	0.630
ypT, per category increase	2.076	1.140–3.780	0.0170	0.616
Combined ypT/ypN model	—	—	—	0.678

The combined ypT/ypN model included ypT category and ypN status as predefined post-NACT pathological variables. Because of the limited number of PFS events, these analyses were intended for parsimonious comparison rather than extensive multivariable adjustment. The original rectal NAR-derived oNAR categories were used only for exploratory visualization and were not interpreted as clinically validated ovarian cancer thresholds. CI—confidence interval; IDS—interval debulking surgery; oNAR—ovarian NAR-like score; NACT—neoadjuvant chemotherapy; PFS—progression-free survival; ypT/ypN—pathological T/N status after IDS.

## Data Availability

The dataset analyzed during the current study is available from the corresponding author upon reasonable request and subject to institutional approval.
